# Supplementary material for: Sorting at embryonic boundaries requires high heterotypic interfacial tension
Source: Nat Commun. 2017 Jul 31;8:157. doi: 10.1038/s41467-017-00146-x (PMC5537356; doi:10.1038/s41467-017-00146-x)
Supplement: Supplementary file 2 — Supplementary Software 1 [file 41467_2017_146_MOESM2_ESM.zip › PottsModel/SrcPottsModel/doc/model/class-use/Coordinates.html]

Uses of Interface model.Coordinates


JavaScript is disabled on your browser.


Skip navigation links


- Overview
- Package
- Class
- Use
- Tree
- Deprecated
- Index
- Help

- Prev
- Next

- Frames
- No Frames

- All Classes

## Uses of Interface model.Coordinates

- Packages that use Coordinates

  | Package | Description |
  | --- | --- |
  |  |  |
  | --- | --- |
  | gui |  |
  | model |  |
- - ### Uses of Coordinates in gui

    Classes in gui that implement Coordinates

    | Modifier and Type | Class and Description |
    | --- | --- |
    |  |  |
    | --- | --- |
    | `class` | `Hexagon.AxialCoordinates` |
    | `class` | `Hexagon.CubeCoordinates` |
    | `class` | `Hexagon.OffsetCoordinates` Even-q vertical layout. |

    Methods in gui that return Coordinates

    | Modifier and Type | Method and Description |
    | --- | --- |
    |  |  |
    | --- | --- |
    | `Coordinates` | Square.`getCoordinates()` |
    | `Coordinates` | PixelShape.`getCoordinates()` |
    | `Coordinates` | Hexagon.`getCoordinates()` |
    | `Coordinates` | Square.`getNeighborCoordinates(PixelShape.Edge pEdge)` |
    | `Coordinates` | PixelShape.`getNeighborCoordinates(PixelShape.Edge pEdge)` |
    | `Coordinates` | Hexagon.`getNeighborCoordinates(PixelShape.Edge pEdge)` |
  - ### Uses of Coordinates in model

    Classes in model that implement Coordinates

    | Modifier and Type | Class and Description |
    | --- | --- |
    |  |  |
    | --- | --- |
    | `class` | `CartesianCoordinates` |

    Methods in model that return Coordinates

    | Modifier and Type | Method and Description |
    | --- | --- |
    |  |  |
    | --- | --- |
    | `Coordinates` | Cell.`getCenterOfMass()` |
    | `Coordinates` | Pixel.`getCoordinates()` |

    Methods in model with parameters of type Coordinates

    | Modifier and Type | Method and Description |
    | --- | --- |
    |  |  |
    | --- | --- |
    | `static FiniteVector` | FiniteVector.`createVectorFromCoordinates(Coordinates c1, Coordinates c2)` |

    Constructors in model with parameters of type Coordinates

    Constructor and Description

    `HalfLine(Coordinates c1,
    Coordinates c2)`

Skip navigation links


- Overview
- Package
- Class
- Use
- Tree
- Deprecated
- Index
- Help

- Prev
- Next

- Frames
- No Frames

- All Classes
